# Posterior uniportal endoscopic laminotomy for cervical ossification of posterior longitudinal ligament: a case report and technical summary

**DOI:** 10.3389/fsurg.2026.1786576

**Published:** 2026-04-20

**Authors:** Baoliang Li, Zhigang Shi, Jianxin Zhang, Nianhu Li, Changjiao Ji

**Affiliations:** 1 Department of Orthopedics, Affiliated Hospital of Shandong University of Traditional Chinese Medicine, Jinan, China; 2 First Clinical Medical College, Shandong University of Traditional Chinese Medicine, Jinan, China

**Keywords:** endoscopic spine surgery, minimally invasive spine surgery, ossification of the posterior longitudinal ligament, posterior endoscopic laminotomy, unilateral radiculopathy

## Abstract

**Background:**

The management of cervical ossification of posterior longitudinal ligament (OPLL) with unilateral radiculopathy poses significant challenges. Posterior uniportal endoscopic laminotomy offers a minimally invasive alternative, yet its application in OPLL-related stenosis remains technically demanding and underreported.

**Case summary:**

A 49-year-old female presented with progressive left upper limb radiculopathy, numbness, weakness, and cervicobrachial pain due to OPLL-induced severe neuroforaminal and lateral recess stenosis at C6-C7 and C7-T1. Through a single 1-cm incision, posterior endoscopic decompression was performed via unilateral laminotomy at both target levels. The procedure was completed in 1.5 h with minimal blood loss. Postoperatively, the patient showed rapid symptomatic improvement, with significant reduction in pain and recovery of grip strength by 6-month follow-up. Integrating contemporary evidence with technical experience, we outline key procedural insights to support the adoption of this technique in selected OPLL cases.

**Conclusion:**

Posterior uniportal endoscopic laminotomy is a feasible and effective minimally invasive option for selected OPLL patients with unilateral radiculopathy. It achieves clinical improvement while preserving spinal motion and avoiding fusion-related complications, provided patient selection and surgical technique are optimized.

## 1 Introduction

Cervical ossification of posterior longitudinal ligament (OPLL) is a common degenerative condition in East Asian populations, with reported prevalence rates of approximately 4.1% in China and 6.3% in Japan (1). It can lead to progressive spinal canal stenosis and compression of neural structures. Clinical manifestations range from asymptomatic presentation to severe myelopathy or radiculopathy, with symptoms including neck pain, limb numbness, motor deficits, and gait disturbances (2). When conservative management fails, surgical intervention becomes necessary to achieve neural decompression and prevent neurological deterioration.

The conventional surgical approaches for cervical stenosis caused by OPLL primarily include anterior procedures—such as anterior cervical corpectomy and fusion (ACCF) and anterior cervical discectomy and fusion (ACDF)—as well as posterior procedures, such as laminectomy and laminoplasty (3). Each approach possesses distinct advantages and limitations regarding the extent of decompression, impact on spinal stability, and associated complication profiles. Notably, anterior techniques carry specific risks, including graft-related complications, dysphagia, hoarseness, and cerebrospinal fluid leakage. Posterior decompressive procedures, though technically less demanding in some respects, may be limited in their efficacy for addressing ventral compression.

The advancements in minimally invasive spine surgery have established endoscopic techniques as a significant alternative for the treatment of degenerative cervical spine conditions. Their core advantages lie in minimizing tissue trauma, preserving spinal motion segments, and facilitating rapid postoperative recovery (4). Among these techniques, posterior endoscopic discectomy decompression has emerged as a highly effective and minimally invasive procedure for the treatment of cervical radiculopathy caused by lateral or paracentral soft disc herniation or foraminal stenosis. Numerous clinical studies have confirmed its favorable efficacy and safety profile (5). Compared with traditional open surgery, posterior endoscopic discectomy techniques demonstrate significant advantages in operative time, incision length, intraoperative blood loss, and hospital stay (6). It significantly improves Visual Analog Scale (VAS) scores for arm and neck pain and the Neck Disability Index (NDI). The degree of improvement is comparable to, if not superior to, that of ACDF, which is considered the “gold standard,” while maintaining low rates of complications and reoperation (7, 8). More importantly, as a non-fusion technique, posterior endoscopic discectomy avoids implant-related complications, preserves motion at the operative segment, and theoretically helps reduce the risk of adjacent segment degeneration. Although endoscopic techniques have become increasingly mature in managing soft disc herniation and ligamentous hypertrophy-related stenosis, their application for cervical canal stenosis caused by ossification of the OPLL remains a significant challenge, with relatively few clinical reports available.

This report discusses a case of cervical OPLL with spinal canal stenosis, where the patient primarily experienced left upper limb muscle weakness, numbness, radicular pain, and cervicobrachial pain. We describe the successful application of a purely posterior uniportal endoscopic laminotomy decompression technique. By integrating contemporary evidence with our technical experience, this report aims to outline critical procedural insights and discuss the potential role of endoscopic laminotomy decompression surgery in the management of select cases of OPLL and spinal canal stenosis.

## 2 Case presentation

A 49-year-old female presented with a 1 year history of progressive severe cervicoscapular pain and left forearm pain (ulnar distribution) and numbness in the ulnar palm and 3th-5th digits. Preoperative NDI was 27 and VAS-arm was 8. She reported significant weakness impacting left-hand grip strength and fine motor dexterity (e.g., buttoning clothes), consistent with C7/C8 radiculopathy. No history of trauma or antecedent neck pain was reported. Physical examination revealed hypoesthesia in the C7/C8 dermatome, reduced left grip strength, no hyperreflexia in all four limbs, and a negative Hoffman's sign, effectively ruling out myelopathy.

**Figure 1:**
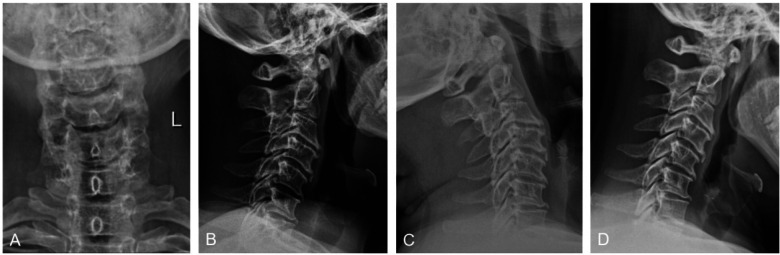
Preoperative cervical x-ray. **(A)** The anteroposterior view of the cervical vertebra demonstrates signs of degeneration, with a loss of normal curvature. **(B)** The lateral view reveals further cervical vertebral degeneration and a straightened physiological curvature. **(C,D)** Dynamic imaging (flexion and extension views) shows no signs of cervical vertebral instability, although degenerative changes are evident in these positions.

**Figure 2:**
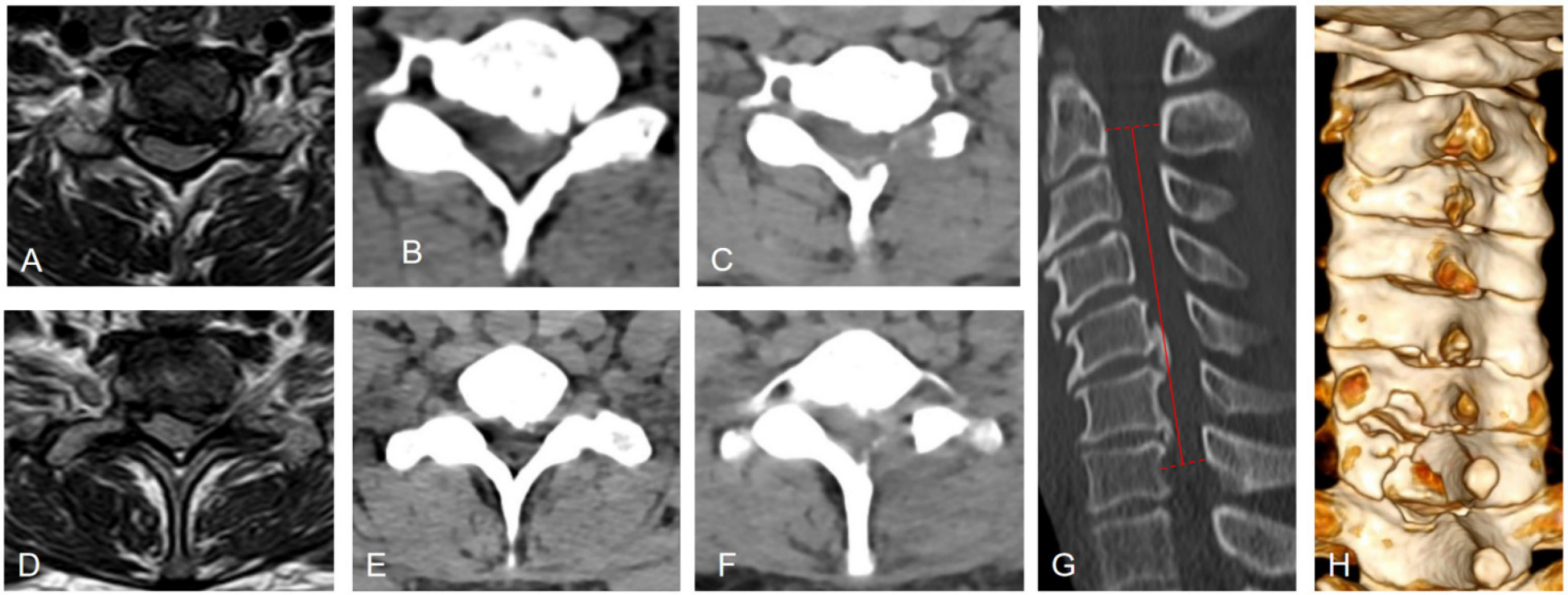
Preoperative and postoperative imaging of C6-C7 and C7-T1 segments. **(A–C)** C6-C7 Segment: **(A)** MRI shows significant spinal canal stenosis, more pronounced on the left side. **(B)** CT scan reveals left-sided ossification of the posterior longitudinal ligament, leading to stenosis of the neural foramen.**(C)** Postoperative imaging confirms adequate decompression. **(D–F)** C7-T1 Segment: **(D)** MRI demonstrates obvious spinal canal stenosis, more severe on the left side. **(E)** CT scan shows less ossification of the posterior longitudinal ligament on the left compared to the C6-C7 segment, with spot-like calcification, indicating ongoing ossification.**(F)** Postoperative imaging shows sufficient decompression. **(G)** Sagittal CT of the cervical spine with the K-line marked, K-line (-). **(H)** Postoperative 3D reconstruction illustrates partial excision of the lamina and preservation of the facet joints, confirming effective decompression.

The anteroposterior, lateral, and dynamic views of the cervical spine demonstrate definite osteophyte formation, straightening of the normal cervical lordosis, and no significant instability on dynamic imaging. Combined MRI and non-contrast CT scans demonstrate significant ossification of the posterior longitudinal ligament (OPLL) at the C6-C7 and C7-T1 levels, resulting in severe spinal canal stenosis. The narrowing is more pronounced on the left side, where there is also marked stenosis of the left neural foramen (Figures 1, 2).

## 3 Surgical procedure

**Figure 3:**
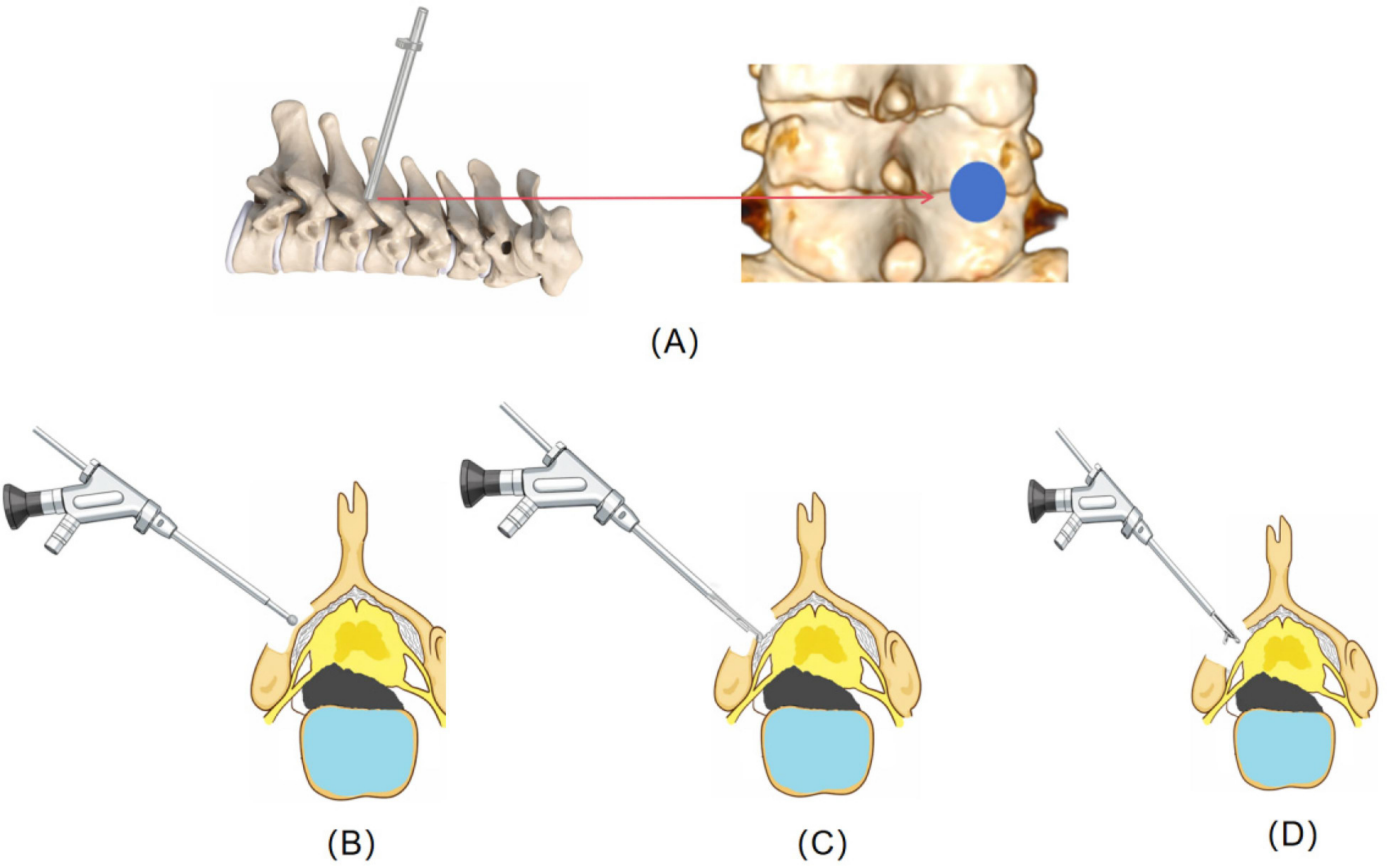
Schematic diagram illustrating the key steps of endoscopic laminotomy. **(A)** The working cannula of the endoscope was positioned at the V-point. **(B)** A high-speed diamond burr was used to initially thin the lamina. **(C)** The remaining wafer-thin cortical bone was then precisely removed using an endoscopic laminectomy punches. **(D)** The ligamentum flavum was resected using a nucleus pulposus forceps.

The procedure was initiated following the induction of general anesthesia. The patient was positioned in the prone position with the neck secured in slight flexion using a head holder. Chest and pelvic bolsters were employed to facilitate positioning. The cervical spine was then imaged using C-arm fluoroscopy to precisely identify the C7-T1 level. A skin incision, approximately 1 cm in length, was made approximately 1.5 cm lateral to the midline, corresponding to the projected line of the facet joints on the anteroposterior view (Figure 3).

Under continuous fluoroscopic guidance, a puncture needle was advanced through the incision until its tip contacted the bony surface at the medial margin of the facet joint complex. This critical anatomical landmark, often referred to as the “V-point” or “Y-point,” represented the junction of the inferior margin of the superior lamina, the superior margin of the inferior lamina, and the medial aspect of the articular process. A guidewire was introduced, followed by sequential dilators to gently spread the soft tissues. A beveled working cannula was then positioned over the final dilator, and its final placement at the V-point was confirmed radiographically before the dilators and guidewire were removed.

The endoscope was introduced into the irrigated working channel, providing direct visualization. Soft tissue overlying the bony surfaces was cleared using bipolar radiofrequency and endoscopic forceps to fully expose the V-point. The cornerstone of the procedure, the creation of the keyhole bony window, was performed centered on this landmark. A high-speed diamond burr was employed to initially thin the bone at the inferolateral portion of the superior lamina and the superolateral portion of the inferior lamina. The remaining wafer-thin cortical bone was then precisely removed using endoscopic laminectomy punches. The medial aspect of the facet joint was partially resected as needed, with care taken to preserve more than 50% of the articular process to maintain postoperative stability. This created an oval laminotomy, the size of which was tailored to the pathology.

Following adequate unroofing, the ligamentum flavum was carefully dissected and resected using nucleus pulposus forceps, exposing the underlying neural structures. The epidural venous plexus was coagulated with bipolar radiofrequency to maintain a clear field. The lateral dura and the exiting nerve root were identified. Adhesions around the nerve root were released with a dissector and radiofrequency. Decompression was deemed complete when the nerve root was visibly relaxed along its course, and the dural sac exhibited free pulsation. The herniated disc anterior to the nerve root and spinal cord, as well as the ossified posterior longitudinal ligament, were not addressed.

Through the same skin incision, the working cannula was slightly repositioned and redirected cephalad. Its trajectory was adjusted to override the superior border of the C7 lamina, thereby transitioning the surgical target to the C6-7 interlaminar space. The subsequent decompression procedure at this level was performed in an identical manner to the previously described technique. After confirming meticulous hemostasis, all instruments were withdrawn under direct vision. The incision was closed in layers without the need for a drain. The procedure resulted in an estimated blood loss of 50 mL and had a total operative duration of 1.5 h.

## 4 Postoperative management and follow-up

Postoperative CT imaging confirmed adequate decompression of the neural elements with the majority of the facet joints preserved (Figure 2). The patient was mobilized with cervical collar protection on postoperative day 1. The collar was worn for 3 days to mitigate discomfort from local surgical trauma. An intravenous dexamethasone regimen was administered over 3 days postoperatively, supplemented by oral non-steroidal anti-inflammatory drugs for analgesia. No complications such as surgical site infection or neurological deterioration were observed, and the patient reported no axial neck pain. At the time of discharge, the VAS score for left upper limb pain was 2, and the NDI score was 13. The patient exhibited a reduction in the extent of left-hand numbness and an improvement in left-hand grip strength compared to preoperative status. Discharge occurred on postoperative day 3 with instructions to perform hand function exercises using a grip strengthener. At the 6-month follow-up, the VAS score for left upper limb pain had improved to 1, and the NDI score was 6. Both the extent and severity of left-hand numbness showed significant improvement, and left-hand grip strength had essentially returned to normal.

## 5 Discussion

The present case exemplifies a classic presentation of symptomatic cervical stenosis secondary to ossification of the OPLL, characterized by unilateral radiculopathy without myelopathic signs, neuroforaminal and lateral recess compression, and loss of cervical lordosis. These features pose challenges for conventional surgical approaches while presenting an opportunity for minimally invasive endoscopic intervention. Traditional surgical strategies for cervical OPLL are primarily divided into anterior decompression and fusion procedures and posterior expansive laminoplasties. Anterior approaches, while enabling direct resection of the ossified ligament, carry risks of dural tear, spinal cord injury, graft-related complications, and approach-related morbidities, particularly in cases with high canal occupancy ratios or severe adhesions (1). Posterior laminoplasty, although achieving indirect decompression, may inadequately address focal lateral or foraminal stenosis and can lead to postoperative axial neck pain, stiffness, or loss of motion segment integrity (9). The patient's pathology, being predominantly unilateral, confined to the neuroforamen and lateral recess, and coupled with maintained cervical lordosis and absence of myelopathy, supports a targeted posterior decompression strategy without the necessity for fusion.

The evolution of cervical endoscopic spine surgery has established posterior endoscopic decompression as a viable treatment for unilateral radiculopathy secondary to soft disc herniation or foraminal stenosis, with clinical success rates exceeding 90% and low reoperation rates (10, 11). The minimally invasive paradigm of endoscopic surgery preserves posterior cervical musculoskeletal structures—including the spinous process, supraspinous and interspinous ligaments—and minimizes soft tissue dissection via a narrow surgical portal, conferring substantial advantages over conventional open techniques. These benefits include shorter hospital stays, with multiple studies confirming that endoscopic approaches reduce inpatient duration. For instance, Akbari et al. reported a mean hospital stay of only 1 day following endoscopic decompression, markedly shorter than the 5–7 days typical of open ACDF (12). Ma et al. further documented a mean hospital stay of 3.7 ± 1.3 days in endoscopic cervical foraminotomy patients, vs. 6.8 ± 1.5 days in ACDF patients (*p* < 0.001) (13). Endoscopic techniques also lower infection risk. In a systematic review by Ju and Lee examining complications in endoscopic spinal surgery, the surgical site infection (SSI) rates of <1% for cervical endoscopic procedures, compared with 2%–4% for open cervical spine surgery (14). A comparative study by Tang et al. of 160 patients undergoing unilateral biportal endoscopic and full-endoscopic foraminotomy found no SSI in either endoscopic cohort (10). Conventional ACDF sacrifices segmental mobility and may accelerate adjacent segment degeneration (ASD) with an incidence of 12%–25%. In contrast, endoscopic techniques preserve facet joints (resection rate < 50%) and avoid fusion, maintaining spinal biomechanical integrity. A study of 61 patients with 5 years of follow-up found that cervical sagittal alignment was maintained or even improved with significantly less postoperative axial neck pain after endoscopic laminotomy, whereas cervical sagittal alignment decreased with more severe axial neck pain after conventional expansive laminoplasty (15). Collectively, these findings indicate that cervical endoscopic surgery offers meaningful clinical advantages—including shorter hospital stays, lower infection rates, and preserved cervical motion—thus reducing the risk of adjacent segment degeneration. However, its application in OPLL-related stenosis remains sparsely reported, largely due to the bony nature of the compressive pathology, the broader extent of compression, and the technical challenges associated with ventral osseous pathology. Nevertheless, technical reports have confirmed the feasibility of endoscopic posterior decompression for multilevel OPLL with encouraging 2-year clinical outcomes (16). In this case, we adapted principles from posterior endoscopic decompression, conceptually aligning with the philosophy of “open-door” laminoplasty—that adequate dorsal decompression can alleviate ventral compression through posterior spinal cord drift, provided cervical lordosis is maintained and the compressive lesion is not excessively rigid or centrally predominant.

Critically, OPLL differs fundamentally from soft disc herniation in its nature and surgical risk profile. While a small annular tear may permit removal of herniated material through a limited corridor, OPLL represents a continuous, often dural-adherent, calcified ventral mass (17). Attempting direct endoscopic resection carries a high risk of dural penetration, spinal cord injury, and incomplete decompression. Therefore, our approach emphasized indirect dorsal decompression—enlarging the lateral recess and neuroforamen via endoscopic laminotomy—rather than resection of the ventral ossification. This strategy is supported by biomechanical and clinical studies indicating that adequate posterior decompression in a well-aligned spine allows for posterior cord drift, mitigating the effect of ventral compression (18). Long-term follow-up studies have shown that endoscopic posterior decompression techniques, such as cervical micro-endoscopic laminotomy, can achieve neurological recovery comparable to laminoplasty while significantly reducing postoperative axial pain and better preserving cervical lordosis (15).

Technically, the procedure relied on precise identification of the “V-point” (the junction of the caudal edge of the superior lamina and the medial aspect of the facet joint) and controlled bony removal using a high-speed diamond burr. Anatomical studies suggest that a technique involving resection of both the superior and inferior laminae allows for more effective exposure of the entire nerve root and its axilla compared to an approach limited to the superior lamina, which is critical for achieving adequate decompression (19). This was followed by meticulous ligamentum flavum resection and nerve root decompression. Preserving more than 50% of the facet joint was prioritized to maintain segmental stability—a key principle emphasized in anatomical and clinical studies on posterior cervical laminotomy (20). Addressing two adjacent levels through a single working channel further underscores the efficiency and minimally invasive nature of the endoscopic approach, consistent with evolving techniques like cervical endoscopic unilateral laminotomy for bilateral decompression applied in selected cases of cervical stenosis (6).

Postoperatively, the patient experienced rapid symptom resolution with significant reduction in upper limb pain, recovery of grip strength, short hospital stay, and absence of complications. This outcome aligns with the high success rates reported for endoscopic posterior cervical decompression in treating radiculopathy (11). Systematic reviews also suggest that for unilateral radiculopathy, minimally invasive posterior laminotomy may offer superior arm pain relief compared to ACDF, while being comparable in terms of NDI improvement, complication rates, and reoperation rates (21). However, it must be emphasized that OPLL cases necessitate stricter patient selection—favoring those with lateralized symptoms, maintained lordosis and absence of cervical instability (22).

Nevertheless, the application of posterior endoscopic laminotomy for OPLL remains in its experiential phase. The major limitation of this study is the relatively short 6-month follow-up duration. Given the slowly progressive nature of OPLL, longer follow-up is required to better assess the long-term efficacy, durability, and potential recurrence of the surgical procedure. Future prospective comparative studies are needed to systematically evaluate its efficacy against anterior decompression and fusion or posterior laminoplasty, particularly regarding the quality of neurological recovery, symptom recurrence rates, long-term cervical stability, and radiographic assessment of ossification progression. Furthermore, advancements in intraoperative navigation, augmented reality, endoscopic visualization, and dedicated instrumentation for bony work may enhance the safety, precision, and potentially broaden the indications for managing such complex osseous pathologies.

## 6 Conclusion

In conclusion, for carefully selected OPLL patients presenting with unilateral radiculopathy and focal lateral recess stenosis, posterior uniportal endoscopic laminotomy represents a feasible and minimally invasive alternative. By employing an indirect decompression strategy based on the principle of posterior cord shift and respecting the unique pathological nature of OPLL, this method can achieve meaningful clinical improvement while avoiding fusion, preserving motion segments, and reducing complications associated with open procedures. Further clinical validation and technical refinement are required to more definitively establish its position within the stepped-care surgical algorithm for cervical OPLL.

## Data Availability

The raw data supporting the conclusions of this article will be made available by the authors, without undue reservation.
